# Unmet Spiritual Needs, Spiritual Wellbeing and Support Satisfaction of End-of-Life Patients: Findings from a Spiritual and Existential Care Training Program (SpECi) for Health Care Practitioners within Inpatient Geriatric Care, Palliative Care Units and Hospices

**DOI:** 10.1007/s10943-025-02273-y

**Published:** 2025-02-18

**Authors:** Arndt Büssing, Marianne Kloke, Mareike Gerundt

**Affiliations:** 1 Faculty of Health, Witten/Herdecke University, Gerhard-Kienle-Weg 4, 59313 Herdecke, Germany; 2 Clinic for Palliative Medicine (Retired), Evangelische Kliniken Essen-Mitte, Essen, Germany

**Keywords:** Spiritual care training, Spiritual needs, Spiritual wellbeing, Support satisfaction, Health care professionals, Patients, Geriatric care, Palliative care, Hospices

## Abstract

The outcomes of a 40-h Spiritual/Existential Care training program (SpECi) for healthcare professionals on their patients' treatment satisfaction (n = 774) were evaluated. Most patients felt supported in their spiritual needs by the staff (79–81%). This satisfaction was highest in hospices and palliative care units (Eta^2^ = .065, *p* < .001). Regression analyses revealed that satisfaction with spiritual needs support was predicted by therapeutic staff support, treatment place, and support from pastoral care providers (R^2^ = .17). The prominent role of healthcare practitioners with their specific competencies and spiritual care ideals is crucial for patients' support satisfaction which was higher for family and staff than to pastoral care providers.

## Background

People at the end of their lives don't just suffer physically, they also have existential and spiritual needs (Balboni et al., 2022; Puchalski et al., 2009). End-of-life suffering thus encompasses the physical, psychological, and social, as well as the spiritual dimension of human beings. It is thus of particular importance for people in their last phase of life (but not only there) that their existential and spiritual struggles, doubts, and needs are considered relevant, too. For the WHO, Palliative Care thus aims to relieve “suffering through the early identification, correct assessment and treatment of pain and other problems, whether physical, psychosocial or spiritual.” (WHO 2020). However, addressing this spiritual side of patients is still a challenge for healthcare professionals (healthcare practitioners) while others see it as important (Curling et al., 2006; Lee & Baumann, 2013; Frick et al., 2021). Reasons to not address this topic in the clinical routine are professional neutrality, lack of time, lack of knowledge, and general discomfort, but also the assumption of not being responsible for this topic (Curling et al., 2006; Lee & Baumann, 2013; Frick et al., 2021). Nevertheless, particularly in secular societies, patients often would like to talk about their spiritual needs with their medical doctors, but less often with chaplains (Büssing et al., 2009a).

Germany can be regarded as an increasingly non-religious society with a significant decline in Church members within the last decades. In 2023, 46% are Christians, 4% Muslims, 4% other, and 46% without a religious denomination (FOWID 2024). Spiritual Care providers thus may face an increasing number of people who may have had Christian socialization but do not consider it as relevant in their lives anymore. This may have an influence on their expectations of whether they may benefit from spiritual care at all, and further on the question of who should be responsible for non-religious patients. Church-related pastoral care providers/chaplains are best trained, but healthcare-related spiritual care providers are closer to patients´ concerns (Büssing, 2024a; Nauer, 2024; Paal & Grabenweger, 2024).

Additional and structured training is strongly recommended to improve spiritual care for each patient in his or her last phase of life. Of course, also untrained healthcare practitioners with sensitivity to the existential and spiritual struggles of their patients may intuitively do the “right things” in their interactions with their patients. Nevertheless, a profound training would help to further develop awareness, attitudes, and practical skills as well. Even without specific training, health cate professionals may have profession inherent Spiritual Care competencies (Frick et al., 2019; Pastrana et al., 2021; Muhammad Soheil et al., 2022) which could be further developed (Büssing et al., 2024). Yet, due to their increasing work burden and job-related stressors, the signs of spiritual distress and unmet spiritual needs can easily be overlooked and overheard.

Spiritual and existential needs are all those expectations and hopes related to finding meaning, support, inner peace, etc. in one´s life. In contrast, spiritual distress addresses all factors and struggles that disturb a person's belief or value system (Büssing, 2021a, 2021b). Therefore, the assessment of patients´ spiritual needs and wellbeing is essential, as these are the concrete reasons for specific reactions – based on the documented needs. The *Spiritual Needs Questionnaire* (SpNQ) as a standardized assessment facilitates the quick identification of unmet existential and spiritual needs (Büssing, 2021a, 2021b; Nissen et al., 2020; Seddigh et al., 2016). These needs can be categorized as religious needs, existential needs, inner peace needs, generativity needs, and as a further optional dimension, family support needs (Büssing et al., 2018; Büssing, 2021a, 2021b). These categories refer to four core dimensions, Connection, Peace, Meaning/Purpose, and Transcendence (Büssing & Koenig, 2010). These categories were theoretically predefined and verified by factor analyses in different samples and various cultures (Büssing, 2021a, 2021b). Depending on culture, specific items may load on the existential needs factor or the inner peace needs factor. Yet, the respective needs are clearly differentiated from religious needs (Büssing, 2021a, 2021b).

Meanwhile, there are several training programs in Spiritual Care available. These may differ in duration and content, cultural background, professional alignment, the target group of participants, and thus in resulting skills and competencies (Bandini et al., 2019; Hu et al., 2019; Jones et al., 2021; Chahrour et al., 2021; Shamsi et al., 2022; Vitorina et al., 2023; Viftrup et al., 2023; Best et al., 2023; Kang et al., 2023; Frick et al., 2023, Giebel et al., 2024, Jones et al., 2024). In Germany, a multi-professional team developed a basic 40-h training program on Spiritual/Existential Care (SpECi) for people from different health professions (Gerundt, 2024; Gerundt et al., 2023; Giebel et al., 2024). To our knowledge, it is the only Spiritual Care program that is accredited by the German Society of Palliative Medicine (Deutsche Gesellschaft für Palliativmedizin) (Giebel et al., 2024).

The aim of this training was to strengthen the Spiritual Care competencies and skills of the staff from inpatient geriatric care, hospices, and palliative care units. The expectation was to thereby increase their awareness and thus their motivation to support the spiritual needs and spiritual wellbeing of their patients. We expected that a short 40-h training program might 1) sensitize participants to the topic, 2) encourage an empathic care ideal, and 3) improve some of their spiritual care skills as an ideal. Nevertheless, the training program was not intended to be a specialized training program for pastoral workers, but as a low-threshold yet well-founded basic training for health care professionals.

The “EPICC Spiritual Care Education Standard” describes how spirituality and spiritual care could be understood for nursing and midwifery practice across Europe (McSherry et al., 2021; van Leeuwen et al., 2021). The respective competencies were described as 1) Intrapersonal spirituality, 2) Interpersonal spirituality, 3) Spiritual care: assessment & planning, and 4) Spiritual care: intervention & evaluation (McSherry et al., 2021; van Leeuwen et al., 2021). While the “EPICC Standard” (McSherry et al., 2021) was designed for spiritual care education in nursing and midwifery, the SpECi program was designed as an interdisciplinary education program for different health care professionals, i.e., nurses, medical doctors, psychologists, social workers, inclusively chaplains (Giebel et al., 2024). The spirituality definition of the SpECi program (Kloke et al., 2024) refers to the consented definition of the European Association for Palliative Care (EAPC) (Nolan et al., 2011).

Referring to the EPICC Standard, the SpECi program´s teaching content was specified and divided into 10 modules to provide basic knowledge and skills (Gerundt, 2024; Gerundt et al., 2023):

1. Approaches and clarifications to gain a basic understanding of spirituality and Spiritual Care in healthcare;
2. Assessment of spiritual needs (i.e., using the Spiritual Needs Questionnaire for documentation) and the recognition of one's spiritual care competencies;
3. Perception of existential concerns;
4. Spiritual and existential communication;
5. Legal framework of Spiritual Care and existential communication;
6. Communication about ultimate questions;
7. Facing loss and grief;
8. Spiritual resources and sources of strength: What gives comfort, and what gives inner peace?
9. Spiritual resources and sources of strength: What gives hope?;
10. Helpful rituals.

The education matrix of these 10 modules, the specific content, and the intended learning aims are extensively described in the validated curriculum (Giebel et al., 2024).

The primary aim of this explorative analysis was to assess the outcomes of the SpECi-trained healthcare professionals on their patients' wellbeing and treatment satisfaction. We focused on patients´ satisfaction with the support of their spiritual needs by the staff. A further aim was to analyze which variables (including treatment place reflecting their health situation) would predict patients´ support satisfaction.

## Methods

### Recruitment of Health Care Professionals

For this interventional study, 85 healthcare professionals were enrolled. They were training in seven centers as described (Gerundt et al., 2023): 1) von Bodelschwinghschen Stiftungen Bethel in cooperation with Klinikum Bielefeld 2) Caritasverband of the Rhein-Erft area in cooperation with St. Vinzenzhaus Cologne; 3) Evangelisches Krankenhaus Alsterdorf; 4) Community of Franciscan sisters in Olpe, 5) Johannesstift Diakonie Berlin; 6) Evangelische Kliniken Essen-Mitte; 7) Theodor Fliedner Stiftung Duisburg (due to work overload they were not trained, but send data of 35 patients), and 8) an additional German-wide Virtual Training Center in Münster (to replace center 7). These trained participants were 37% nursing, 10% elderly care, 24% social work/social service, 4% pastoral care, 4% voluntary hospice work, and 22% other professions.

They were expected to recruit 10 patients and residents they care for in their respective workplaces per phase of the study. Unfortunately, the study would fall into the COVID-19 pandemic. The first recruitment phase was before the SpECi training program and during the second half of 2021 with the 4th wave of the COVID-19 pandemic, while the training in the respective centers started in winter 2021 and lasted until Spring 2022. The second wave of recruitment started three months after the training during the summer of 2002 with the pandemic´s summer wave, and the third recruitment wave six months later during the harvest and winter wave of the pandemic.

Since the study fell into the aforementioned infection waves of the COVID-19 pandemic, we lost several of the healthcare practitioners to follow-up: 85 participated in recruitment phase one, 48 in phase two, and 39 in phase three. Several of them were infected by themselves or had to cope with increasing workload due to coronavirus-related staff absences, changed their employer, or abandoned their job.

### Recruitment of Patients and Residents

All in all, 774 patients and home residents were enrolled in three consecutive recruitment phases by healthcare practitioners. Due to the loss of participants in the last two phases of the study, the number of enrolled patients and residents decreased significantly, too: 436 were present in recruitment phase one, 199 in phase two, and 139 in phase three.

Inclusion criteria for patients and home residents were the ability to read and respond to the questionnaire (with assistance by the trained staff when needed), informed consent, and an assumed life expectancy of less than 12 months but at least 1 month; dementia (except mild forms) was an exclusion criterion.

### Informed Consent

All participants (healthcare practitioners, patients, and residents) consented to participate and signed an Informed Consent Statement. The study was approved by the Ethical Commission of the University Witten/Herdecke (#S-280/2020).

### Measures

Patients and residents responded anonymously to standardized questionnaires (if required they could rely on help to fill them) once during their stay, and thus no individual time courses could be assessed.

Apart from sociodemographic and basic health-related data, the following instruments were used:

#### Spiritual Needs

To assess participants´ unmet psychosocial, existential, and spiritual needs, the *Spiritual Needs Questionnaire* (SpNQ) was used (Büssing et al., 2018; Büssing, 2021a, 2021b). For this study, an extended 25-item version was applied that addresses the four main dimensions, and additionally Family Support needs.

1. Religious needs (Cronbach´s alpha = 0.87; Cronbach´s alpha = 0.88 in this sample), i.e., praying for and with others, and by themselves, participating in a religious ceremony, reading spiritual/religious books, turning to a higher presence (i.e. God, angels, etc.)
2. Existential needs (Cronbach´s alpha = 0.74; Cronbach´s alpha = 0.80 in this sample), i.e., dissolve open aspects of life, talk with someone about meaning in life/suffering, dissolve open aspects in life, talk about the possibility of life after death, forgive someone from a distinct period of your life
3. Inner Peace needs (Cronbach´s alpha = 0.73; Cronbach´s alpha = 0.65 in this sample), i.e., wish to dwell at places of quietness and peace, plunge into the beauty of nature, find inner peace, talk with others about fears and worries
4. Giving / Generativity needs Cronbach´s (alpha = 0.71; Cronbach´s alpha = 0.65 in this sample) addresses the active and autonomous intention to give solace to someone, to pass own life experiences to others, give away something from yourself, be assured that your life was meaningful and of value
5. Family Support needs (Cronbach´s alpha = 0.69 in this sample) address the need for more support from the family, feeling connected with the family, having more time together, and having someone who provides hope (someone who assures how things or the situation will continue in a positive sense)

The intensity of these needs is scored on a 4-point scale ranging from 0 – not at all; 1 – somewhat; 2 – moderate; 3 – strong. The consented 20 items form the SpNQ-20 mean score (Büssing, 2021a, 2021b) which has a good internal consistency in this sample (Cronbach´s alpha = 0.88) was additionally used to make international data better comparable.

#### Spiritual Wellbeing

Participants´ spiritual wellbeing was assessed with the 12-item *Functional Assessment of Chronic Illness Therapy Spirituality Wellbeing* (FACIT-Sp) scale (Bredle et al., 2011; Peterman et al., 2002). It differentiates three dimensions (Cronbach´s alpha = 0.81–0.88):

1. Meaning (Cronbach´s alpha = 0.63 in this sample): have a reason for living; life has been productive; purpose in life; life lacks meaning and purpose
2. Peace (Cronbach´s alpha = 0.74 in this sample): feel peaceful; trouble feeling peaceful, feel comfort; harmony with myself
3. Faith (Cronbach´s alpha = 0.86 in this sample): find comfort in faith; find comfort in faith; difficult times have strengthened spiritual beliefs; whatever happens with illness, things will be ok

The scoring ranges from 0 – not at all; 1 – a little bit; 2 – somewhat; 3 – quite a bit to 4 – very much. Two items have a reverse scoring. The scores of all 12 statements will represent the mean FACIT-Sp score (Cronbach´s alpha = 0.83 in this sample).

#### Psychological Wellbeing

Psychological wellbeing was assessed with the 5-item *WHO-Five Wellbeing Index* (WHO-5) (Bech et al., 2013). The representative items are “I have felt cheerful and in good spirits” or “My daily life has been filled with things that interest me.” The intensity of feelings refers to the last two weeks and was scored with a 6-step grading scale ranging from 0 – at no time to 5 – all the time” (5). Scores < 13 may indicate low wellbeing. The internal consistency of the scale is good in this sample (Cronbach´s alpha = 0.85).

#### Support Satisfaction

Satisfaction with the support through different resources was measured with the support module of the *Brief Multidimensional Life Satisfaction Scale* (BMLSS) (Büssing & Baumann, 2023; Büssing et al., 2009b). With four items it addresses the satisfaction with the support of family, friends and acquaintances, therapeutic staff (nurses, medical doctors, psychologists, etc.), and pastoral workers, ranging from dissatisfaction to satisfaction (0 – very dissatisfied; 1 – dissatisfied; 2 – mostly dissatisfied; 3 – mixed (about equally satisfied and dissatisfied); 4 – mostly satisfied; 5 – satisfied; 6 – very satisfied). The support satisfaction sum score was referred to as a 100% level (transformed scale score). The 4-items´ internal consistency in this sample is acceptable (Cronbach´s alpha = 0.60).

Additionally, participants were asked whether they have felt supported by the therapeutic team or individual members of the team concerning their spiritual needs as mentioned in the SpNQ during the last two weeks. Response options to perceived support were 1 – no, not at all, 2 – rather no, 3 – yes, somewhat, and 4 – yes, very much.

#### Health Situation

Two numeric rating scales (NRS) were used to assess the self-perceived health situation and impairment (“Please mark on the scale how you would describe your current state of health”; “Please mark on the scale how severely you are affected by your life situation/illness”) on a scale ranging from 0—very bad to 100—excellent and 0—not at all to 100—unbearable, respectively.

### Statistical Analyses

Descriptive statistics are presented as frequencies for categorical variables and as means (± standard deviation, SD) for numerical variables. Reliability analyses (Cronbach´s alpha) and one-way analysis of variance on ranks (Kruskal–Wallis-Test for non-normally distributed variables) were computed with SPSS 28.0.

Given the exploratory character of this study and the various tested variables, we set a stricter significance level at *p* < 0.001. Concerning classifying the strength of the observed correlations, we adjusted the thresholds to r > 0.5 as a strong correlation, an r between 0.3 and 0.5 as a moderate correlation, an r between 0.2 and 0.3 as a weak correlation, and r < 0.2 as negligible or no correlation. For group differences, Eta^2^ values < 0.06 are considered as small effects, between 0.06 and 0.14 as moderate, and > 0.14 as strong.

## Results

### Description of the Sample

The majority of recruited patients and residents (n = 774) were female 75%, widowed (51%), had a Christian denomination (82%), and were living in old/nursing homes (58%), geriatrics/hospitals (14%), palliative care units (13%), or hospice (14%) (Table 1). 34% had cancer as a primary disease, 22% heart-related diseases, 9% lung diseases, 12% metabolic diseases, and various other health impairments/diseases (incl. 5% fractures/surgery, 3% old age, 3% mild dementia, 3% strokes). Their mean age was 80 ± 11 [29–102] years.

**Table 1:** Descriptions of patients and residents recruited as three consecutive samples

		Phase 1	Phase 2	Phase 3	All participants	*p*-value
All participants	n	436	199	139	774	
	%	100.0	100.0	100.0	100.0	
*Gender*						.007
Women	%	70.3	80.4	80.1	74.7	
Men	%	29.7	19.6	19.9	25.3	
*Living situation*						.002
Nursing home	%	57.8	53.9	65.1	58.1	
Geriatrics/Hospitals	%	14.1	18.8	7.8	14.2	
Palliative care unit	%	15.7	8.9	11.6	13.3	
Hospice	%	12.4	16.8	13.2	13.7	
*Religious denomination*						.505
Christian	%	81.8	82.2	83.5	82.2	
Other	%	3.3	1.0	0.8	2.3	
None	%	14.9	16.8	15.7	15.5	
Mean age (years)		79.9 ± 11.0	80.4 ± 11.6	80.5 ± 12.0	80.1 ± 11.3	.799
Health situation (NRS)		47.2 ± 21.2	45.6 ± 20.7	50.2 ± 21.7	47.3 ± 21.2	.138
Perceived impairment (NRS)		56.1 ± 24.0	56.6 ± 23.8	55.3 ± 22.6	56.1 ± 23.7	.878

There were no significant differences in patients´ age or religious denomination within the three samples (recruitment phases), while the proportion of men was significantly lower in phases two and three as compared to phase one; the proportion of patients in Hospice and Geriatrics/Hospitals was higher, and that of patients and residents in Palliative care units and Nursing homes were lower in phase two (Table 1). Neither the self-assessed health situation nor perceived impairment significantly differed in the three samples (Table 1).

### Spiritual Needs, Spiritual and Emotional Wellbeing

Enrolled patients and residents scored high on Family support needs, Inner peace needs, and Generativity needs, followed by Religious needs, and were lowest for Existential needs (Table 2). The Family support needs showed a slight but significant drop in phase two.

**Table 2:** Spiritual needs, Spiritual and Emotional Wellbeing of patients and residents in the three samples

		Spiritual needs (SpNQ)						Spiritual Wellbeing (FACIT-Sp)				Emotional Wellbeing (WHO-5)
		Religious Needs	Existential Needs	Inner Peace Needs	Generativity Needs	Family Support Needs	SpNQ-20 Mean Score	Meaning	Peace	Faith	FACIT-Sp mean score	Emotional wellbeing
All participants	Mean	1.24	0.87	1.75	1.75	1.83	3.14	3.14	2.61	2.19	2.65	12.18
	SD	0.96	0.74	0.77	0.75	0.79	0.76	0.76	0.91	1.23	0.75	6.15
Phase 1	Mean	1.24	0.85	1.77	1.77	1.89	3.14	3.14	2.60	2.16	2.63	12.35
	SD	0.95	0.73	0.77	0.76	0.78	0.78	0.78	0.93	1.26	0.79	6.14
Phase 2	Mean	1.19	0.86	1.74	1.76	1.70	3.09	3.09	2.66	2.23	2.66	12.18
	SD	0.92	0.70	0.78	0.69	0.76	0.77	0.77	0.86	1.20	0.71	6.07
Phase 3	Mean	1.32	0.98	1.71	1.68	1.84	3.19	3.19	2.59	2.23	2.67	11.65
	SD	1.04	0.82	0.76	0.79	0.83	0.70	0.70	0.89	1.18	0.68	6.33
Kruskal–Wallis-H		0.98	2.41	0.76	1.25	9.64	0.24	1.59	0.51	0.50	0.10	1.57
Eta2 value		.009	.001	.001	.002	.018	.002	.004	.001	.001	.002	.008
*p*-value		n.s	n.s	n.s	n.s	.008	n.s	n.s	n.s	n.s	n.s	n.s

Concerning their spiritual wellbeing, the Meaning dimension scored highest, followed by Peace and Faith; yet, there were no significant differences between the three samples (Table 2). Also for emotional wellbeing, there were no significant differences in the three samples (Table 2).

### Support Satisfaction

All in all, 45% of patients and residents felt that their spiritual needs were supported very well by the healthcare practitioners, 34% felt somewhat supported, 12% felt rather not that well supported, and 9% said that their spiritual needs were not at all supported. The spiritual support satisfaction was somewhat higher in phase three, but not significantly (Table 3). In phase one, 45% felt very well supported and 32% somewhat supported (77%), in phase two, 43% felt very well supported and 38%w somewhat supported (81%), and in phase three, 47% felt very well supported and 34% somewhat supported (81%). However, this slight increase is not statistically significant.

**Table 3:** Support satisfaction of patients and residents in the three samples

		Support Satisfaction (BMLSS-Support)					Support of spiritual needs by the staff
		Family	Friends and Acquaintances	Therapeutic staff	Pastoral workers	Mean score	
All participants	Mean	4.88	4.16	4.84	3.74	74.89	3.14
	SD	1.49	1.64	1.18	1.62	17.16	0.96
Phase 1	Mean	4.91	4.16	4.87	3.68	74.87	3.12
	SD	1.51	1.63	1.19	1.68	17.35	0.97
Phase 2	Mean	4.81	4.12	4.76	3.73	74.06	3.13
	SD	1.49	1.68	1.20	1.56	17.54	0.97
Phase 3	Mean	4.87	4.24	4.84	3.97	76.16	3.22
	SD	1.44	1.64	1.13	1.48	15.99	0.90
Kruskal–Wallis-H		1.58	0.52	2.52	2.92	1.05	0.76
*p*-value		n.s	n.s	n.s	n.s	n.s	n.s

The satisfaction with the support of their spiritual needs differs for the treatment site and living situation (e.g. the places where patients were living or were treated) (*p* < 0.001; Chi^2^). In Nursing homes, 35% felt very well supported and 40% somewhat supported (75%); in Geriatrics/Hospitals, 36% felt very well supported and 31% somewhat supported (67%); in palliative care units, 60% felt very well supported and 22% somewhat supported (82%); in Hospices, 72% felt very well supported and 23% somewhat supported (95%).

The highest general support satisfaction was ascribed to the family and the therapeutic staff, followed by friends and acquaintances, and the lowest was attributed to pastoral workers. There were no significant differences in participants´ support satisfaction in the three samples (Table 3).

### Influence of Treatment Places on Spiritual Needs, Support Satisfaction, and Wellbeing

The indicators of wellbeing showed significant differences in the places and settings where the patients were cared for (Table 4). Psychological wellbeing was lowest in palliative care units and highest in nursing homes, while perceived impairment was highest in hospices and lowest in nursing homes. These group differences are moderate and strong, respectively (Table 4).

**Table 4:** Intensity of spiritual needs and support satisfaction in patients from different living/treatment areas

		Perceived Impairment	Emotional wellbeing	Religious Needs	Existential Needs	Inner Peace Needs	Generativity Needs	Family Support Needs	SpNQ-20 Mean Score	Support of spiritual needs by the staff	Support satisfaction
All participants	Mean	55.87	12.19	1.24	0.87	1.76	1.75	1.83	1.33	3.12	74.97
	SD	23.67	6.15	0.96	0.74	0.77	0.75	0.79	0.61	0.96	17.14
Nursing home	Mean	48.84	13.66	1.39	0.78	1.64	1.72	1.73	1.33	3.01	72.58
	SD	22.97	5.81	0.96	0.71	0.73	0.76	0.82	0.61	0.94	16.93
Geriatrics/Hospitals	Mean	59.89	10.94	0.94	0.89	1.83	1.68	2.07	1.25	2.86	73.19
	SD	23.22	6.49	0.91	0.74	0.77	0.69	0.71	0.62	1.10	17.51
Palliative care unit	Mean	67.77	9.06	0.98	1.10	1.90	1.82	1.92	1.37	3.34	79.05
	SD	21.28	5.53	0.94	0.78	0.83	0.75	0.73	0.62	0.97	16.83
Hospice	Mean	70.00	10.30	1.12	1.01	2.04	1.89	1.91	1.42	3.64	82.98
	SD	16.54	5.93	0.92	0.78	0.74	0.77	0.75	0.59	0.69	14.85
Kruskal–Wallis-H		115.74	64.35	33.81	19.11	29.39	6.14	18.66	4.00	52.22	44.07
Eta2 value		.139	.087	.041	.026	.039	.008	.026	.006	.065	.051
*p*-value		< .001	< .001	< .001	< .001	< .001	.105	< .001	.261	< .001	< .001

For the intensity of spiritual needs, there were significant differences between the treatment/living areas (Table 4). Religious needs were highest in residents from nursing homes, Existential needs were highest in patients from palliative care units, Inner Peace needs were highest in hospices, and Family Support needs were highest in geriatrics/hospitals. However, there were no significant differences for Generativity needs.

Spiritual wellbeing in terms of the Meaning component showed no significant difference for the treatment/living areas (Eta^2^ = 0.003), while Peace (Eta^2^ = 0.027) and Faith (Eta^2^ = 0.023) were differently expressed (*p* < 0.001) (data not shown).

Support satisfaction was highest in patients from hospices. Specifically, support of spiritual needs by the staff was highest in hospices and lowest in geriatrics/hospitals, with a moderate effect size (Table 4).

### Correlation and Regression Analyses

To address whether the unmet spiritual needs depend on patients´ perceived impairment and low emotional or spiritual wellbeing, correlation analyses were performed (Table 5). Perceived impairment was weakly related to Existential needs and Inner Peace needs, but not to Religious needs, and marginally only to Generativity needs and Family Support needs. A similar pattern was found for psychological wellbeing. However, Existential needs were weakly negatively related to the spiritual wellbeing component of Peace, but not to Meaning. Inner Peace needs were marginally negatively related to the spiritual wellbeing component of Peace. In contrast, Religious needs were strongly positively related to the spiritual wellbeing domain of Faith. The extent of spiritual needs was either not at all or marginally only related to participants´ general support satisfaction and satisfaction with the staff´s support of spiritual needs (Table 5). Support satisfaction was weakly related to the Meaning and the Faith component, while the perceived support of spiritual needs by the staff was marginally only related to Faith, but not to Meaning or Peace.

**Table 5:** Correlation between spiritual needs, spiritual wellbeing, and support satisfaction

	Religious Needs	Existential Needs	Inner Peace Needs	Generativity Needs	Family Support Needs	SpNQ-20 Mean Score	Support satisfaction	Support of spiritual needs
Health situation	.071	− .133**	− .116**	− .009	− .103**	− .046	.024	−.025
Perceived Impairment	− .031	.217**	.216**	.100**	.150**	.145**	.016	.067
Psychological wellbeing	.078	− .208**	− .196**	.018	− .188**	− .088	.161**	.067
Religious Needs	1.000						.123**	.051
Existential Needs	.371**	1.000					.028	.071
Inner Peace Needs	.294**	.559**	1.000				.132**	.103**
Generativity Needs	.355**	.479**	.465**	1.000			.127**	.123**
Family Support Needs	.243**	.487**	.459**	.394**	1.000		.027	.031
SpNQ-20 Mean Score	.761**	.787**	.704**	.698**	.507**	1.000	.136**	.109**
Meaning	.130**	− .030	.030	.220**	.028	.112**	.272**	.088
Peace	.088	− .257**	− .169**	.019	− .215**	− .088	.186**	.086
Faith	.643**	.116**	.084	.193**	.027	.403**	.233**	.133**
FACIT-Sp mean score	.436**	− .046	− .011	.190**	− .054	.226**	.291**	.130**
Support satisfaction	.123**	.028	.132**	.127**	.027	.136**	1.000	
Support of spiritual needs	.051	.071	.103**	.123**	.031	.109**	.344**	1.000

**p < .001 (Spearman rho)

As there were several variables with a significant influence on patients´ support satisfaction, stepwise regression analyses were performed (Table 6). General Support Satisfaction as a dependent variable was best explained by the treatment place (palliative care units/hospice), by the spiritual wellbeing domain Faith (both explaining 10% of the variance), and further by psychological wellbeing, Inner Peace needs, and Existential needs as a negative predictor with marginal relevance. These five predictors explain together 14% of the variance. Without significant influence in the regression model were Religious needs, Generativity needs, Spiritual wellbeing: Peace, gender, and age.

**Table 6:** Predictors of patients´ general support satisfaction and satisfaction with the support of their spiritual needs (stepwise regression analyses)

Dependent variable: Support Satisfaction Model 5: F = 23.5, *p* < .001; R^2^ = .140		Beta	T	*p*-value
	(Constant)		16.432	< .001
	Spiritual wellbeing: Faith	.185	5.028	< .001
	Treatment side: Palliative care unit / Hospice	.257	7.151	< .001
	Psychological wellbeing	.180	4.730	< .001
	Inner Peace needs	.147	3.558	< .001
	Existential needs	− .084	− 2.017	.044

Dependent variable: Support of spiritual needs Model 6: F = 25.9, *p* < .001; R^2^ = .197		Beta	T	*p*-value
	(Constant)		5.089	< .001
	Support from the Therapeutic Staff	.286	7.348	< .001
	Treatment side: Palliative care unit / Hospice	.118	3.015	.003
	Support by Pastoral Care provider	.101	2.608	.009
	Generativity needs	.075	2.067	.039
	Spiritual wellbeing: Faith	.090	2.363	.018
	Age	− .088	− 2.316	.021

Only significant variables were depicted

Concerning patients' perceived support of their spiritual needs by the team, regression analyses revealed that their satisfaction with the support was explained best by Support from the Therapeutic Staff (explaining alone 14% f variance). Further relevant influences were the treatment place and Support by the Pastoral Care provider (explaining an additional 2% of variance), and to some weak extent also Generativity needs, Faith as a spiritual wellbeing component, and younger age (Table 6). Existential needs, Inner Peace needs, psychological wellbeing, and gender were not of relevance in this regression model which would explain 20% of the variance.

## Discussion

The SpECi intervention was planned before the pandemic, and the healthcare practitioners who intended to participate were trained during the 4th wave of the COVID-19 infection in Germany. Their increasing work stress was further aggravated by the outcomes of the pandemic in the respective clinics and workplaces (Büssing et al., 2024). Several of the trained participants were lost during the second and third phases of the study. They were infected with the COVID-19 virus by themselves, or had to cope with the additional burden of work due to coronavirus-related staff absences, changed employers, or abandoned their jobs. Such effects were described for the German hospital staff by others, too (Gräske et al., 2021; Karagiannidis et al., 2021).

Nevertheless, it seems their ideal to also care for their patients´ spiritual needs and wellbeing was not diminished despite their work burden. Several of the addressed Spiritual Care competencies significantly improved after the intervention, while other competencies were high already at the start of the intervention (Büssing 2024b). This means, that even when the staff was burdened by the consequences of the pandemic and high work burden, their Spiritual Care ideals were still high. Similarly, nursing students from Austria regarded their spiritual care competencies, as measured with the SCCS, as high also during the first wave of the COVID-19 pandemic in 2020 – even when their spiritual wellbeing was low at that time (Brandstötter et al., 2021). As a consequence of participants´ intentions and engagement in our study, their patients stated that they felt well supported (on average 79%). In another study, relatives of patients dying in hospitals, nursing homes, or hospices reported that despite the difficult circumstances due to the COVID-19 pandemic-related restrictions, their dying family members received good emotional support from the staff (Büssing & Baumann, 2023). Those healthcare practitioners who were still at work seemed to have full-heartedly cared for their patients, which is indicated by high support satisfaction. As this support satisfaction was high in the current study also before the training, one may consider two possible explanations: 1) Their spiritual care intentions and openness towards this topic might been high because they invested their time for the 40 h SpECi training program (self-selection of motivated people). 2) They had the opportunity to use the SpNQ as an assessment tool and could directly react to the documented spiritual needs as best as they could. Both motivation and communication might influence how they interact with the patients. Therefore, their patients noticed this interest in their spiritual needs and concerns and felt well supported.

Nevertheless, there was no significant increase in patients´ support satisfaction after the training (77% satisfaction before the training and 81% after the training). Further, their spiritual needs and spiritual wellbeing did not change. A prior study with patients from a palliative care unit showed that neither patients´ spiritual needs nor their spiritual wellbeing changed within their 2-week palliative care unit stay (Büssing et al., 2020). This means, that such a short stay cannot significantly change their unmet needs as the health situation is not changing for the better, too. Nevertheless, also these patients felt well supported in their needs (Büssing et al., 2020).

In the current study, healthcare practitioners enrolled the patients they cared for in three consecutive samples within nursing homes, geriatrics/hospitals, palliative care units, and hospices. The indicators of spiritual burden (high spiritual needs and low spiritual wellbeing) were known to the health care practitioners. Therefore, this knowledge could be used as a concrete reason to talk with the patients about their needs and struggles. It is worth underlining patients´ high support satisfaction, despite the difficult situation with the pandemic-related visiting restrictions. They expressed high satisfaction with the support of the family and the staff. However, they felt less supported by pastoral caregivers. This finding is surprising, as pastoral caregivers are those professionals who are best trained to provide spiritual support, guidance, and counseling. Moreover, they are therefore regarded to be the group responsible for addressing patients´ spiritual and religious needs (Kirchoff et al., 2021; Sinclair & Chochinov, 2012). To explain it, one could assume that pastoral care providers may have had restricted access to the treatment sides during the pandemic-related restrictions. Another option could be that the support they provided was timely and emotionally less intensive as compared to the staff who are more frequently available. Nevertheless, regression analyses showed that the best predictors of patients´ satisfaction with the support of their spiritual needs were therapeutic staff, the treatment place, and pastoral care providers. Perceived impairment and health situation were not related to this support satisfaction.

A further important finding from this study is that wellbeing of patients/residents was different in the respective treatment places. Further, also patients´ spiritual needs and spiritual wellbeing scores differed. Some of these spiritual needs were stronger depending on the patient´s situation and mood states (Büssing, 2021b). In this study, perceived impairment and spiritual needs were significantly higher in patients treated in palliative care units and hospices as compared to patients from geriatrics, hospitals, and nursing homes. However, these spiritual needs and the spiritual wellbeing dimensions are not as closely related as one may have expected. Both are more or less independent dimensions with their specific meaning – and this may have consequences for the spiritual care support plans. Even when patients are satisfied with the support of their spiritual needs by the caring staff, they still may have unmet needs that remain vital. One may assume that these still existing spiritual needs may indicate a persistent longing for life – even if life seems to be becoming less.

## Limitations

The healthcare practitioners were trained under the restricted conditions of the COVID-19 pandemic, and several of them were lost to follow-up. Further, concerning the patients, this study is not a longitudinal study that assesses the development of their needs and wellbeing, Instead it relies on data from three consecutive samples recruited by the trained healthcare practitioners at their facilities. At least we can prove that the basic variables remained stable in these three samples (phases of the study). Nevertheless, longitudinal studies are needed on whether patients' spiritual needs and wellbeing can significantly change at all. A recent observation study with patients from a palliative care unit did not find changes in spiritual needs or spiritual wellbeing (Büssing et al., 2020).

We did not assess the concrete interventions provided by the healthcare practitioners in response to the documented spiritual needs and wellbeing indicators. Instead, we asked for patients' self-perceived satisfaction with the support received by the caring team, pastoral workers, friends and acquaintances, and their families.

It might be that our sample of healthcare practitioners consisted of highly motivated participants. Their employers gave them paid time off for this training. At the end of the SpECi course, they received a participation certificate from the German Society for Palliative Medicine (DGP), but no further incentives.

Pastoral care providers are the best-trained group to address patients´ spiritual needs and concerns. The SpECi program does not claim to be able to impart their profound knowledge and skills to healthcare practitioners with their expertise and duties so that they can replace them. It's more about sensitizing healthcare practitioners to this topic and imparting basic skills that may encourage them to react to these needs as the first instances of perception and to take these issues seriously.

The FACIT-Sp scale has its limitations as the generic Meaning and the Peace subscales capture the cognitive and affective aspects of non-religious spirituality, respectively, while the Faith subscale addresses religious issues, but relates to the experience of illness and is therefore contextual. Moreover, both non-religious subscales are contaminated with psychological wellbeing items (Koenig & Carey, 2024) and may thus be falsely positively related to psychological wellbeing. In this sample, the Faith domain is positively related to Religious needs, and may thus indicate that the resource is available rather than a lack of religious wellbeing. In contrast, Existential needs and Inner Peace needs are inversely related to Meaning and Peace, and therefore indicate a lack of meaning and peace, as expected. A similar association was found also in a sample of palliatively treated paints from Germany, underlying the pitfalls of the FACIT-Sp scale particularly in secular societies (Büssing et al., 2020).

## Conclusions

Healthcare practitioners from inpatient geriatric care, palliative care units, and hospices participated in a 40 h SpECi training. We found that the patients they cared for stated a high satisfaction with the support they received from the staff, particularly concerning their unmet spiritual needs. When these motivated healthcare practitioners participate in a basic spiritual/existential care training program (SpECi), they can further develop their competencies and skills to respond to their patients' needs. However, we did not see a further increase in their patients´ high support satisfaction. The findings underline that healthcare practitioners have to consider significant differences in their patients' spiritual needs and wellbeing depending on the underlying health situation, perceived impairment, and thus treatment place. The prominent role of healthcare practitioners with their specific competencies and spiritual care ideals are crucial for patients' support satisfaction which was higher for family and staff as compared to pastoral care providers.

## Data Availability

Not applicable.
